# Peritoneal sepsis caused by *Escherichia coli* triggers brainstem inflammation and alters the function of sympatho-respiratory control circuits

**DOI:** 10.1186/s12974-024-03025-7

**Published:** 2024-02-08

**Authors:** Gjinovefa Kola, Caitlyn W. Clifford, Cara K. Campanaro, Rishi R. Dhingra, Mathias Dutschmann, Frank J. Jacono, Thomas E. Dick

**Affiliations:** 1 Division of Pulmonary, Critical Care and Sleep Medicine, Department of Medicine, Case Western Reserve University, 10900 Euclid Avenue, BRB 319, Cleveland, OH 44106-1714 USA; 2 Division of Pulmonary, Critical Care and Sleep Medicine, Department of Medicine, Louis Stokes Cleveland VA Medical Center, Cleveland, OH 44106 USA; 3 Department of Neurosciences, Case Western Reserve University, Cleveland, OH 44106 USA

**Keywords:** Sepsis, Systemic inflammation, Brainstem, Sympatho-respiratory network, Hypoxia

## Abstract

**Background:**

Sepsis has a high mortality rate due to multiple organ failure. However, the influence of peripheral inflammation on brainstem autonomic and respiratory circuits in sepsis is poorly understood. Our working hypothesis is that peripheral inflammation affects central autonomic circuits and consequently contributes to multiorgan failure in sepsis.

**Methods:**

In an *Escherichia coli* (*E. coli*)–fibrin clot model of peritonitis, we first recorded ventilatory patterns using plethysmography before and 24 h after fibrin clot implantation. To assess whether peritonitis was associated with brainstem neuro-inflammation, we measured cytokine and chemokine levels in Luminex assays. To determine the effect of *E. coli* peritonitis on brainstem function, we assessed sympatho-respiratory nerve activities at baseline and during brief (20 s) hypoxemic ischemia challenges using in situ-perfused brainstem preparations (PBPs) from sham or infected rats. PBPs lack peripheral organs and blood, but generate vascular tone and in vivo rhythmic activities in thoracic sympathetic (tSNA), phrenic and vagal nerves.

**Results:**

Respiratory frequency was greater (*p* < 0.001) at 24 h post-infection with *E. coli* than in the sham control. However, breath-by-breath variability and total protein in the BALF did not differ. IL-1β (*p* < 0.05), IL-6 (*p* < 0.05) and IL-17 (*p* < 0.04) concentrations were greater in the brainstem of infected rats. In the PBP, integrated tSNA (*p* < 0.05) and perfusion pressure were greater (*p* < 0.001), indicating a neural-mediated pathophysiological high sympathetic drive. Moreover, respiratory frequency was greater (*p* < 0.001) in PBPs from infected rats than from sham rats. Normalized phase durations of inspiration and expiration were greater (*p* < 0.009, *p* < 0.015, respectively), but the post-inspiratory phase (*p* < 0.007) and the breath-by-breath variability (*p* < 0.001) were less compared to sham PBPs. Hypoxemic ischemia triggered a biphasic response, respiratory augmentation followed by depression. PBPs from infected rats had weaker respiratory augmentation (*p* < 0.001) and depression (*p* < 0.001) than PBPs from sham rats. In contrast, tSNA in *E. coli*-treated PBPs was enhanced throughout the entire response to hypoxemic ischemia (*p* < 0.01), consistent with sympathetic hyperactivity.

**Conclusion:**

We show that peripheral sepsis caused brainstem inflammation and impaired sympatho-respiratory motor control in a single day after infection. We conclude that central sympathetic hyperactivity may impact vital organ systems in sepsis.

**Supplementary Information:**

The online version contains supplementary material available at 10.1186/s12974-024-03025-7.

## Introduction

Peritonitis can lead to sepsis, a life-threatening organ dysfunction due to a dysregulated host response to infection [1–3]. The beneficial innate immune response to infection can become harmful and cause sepsis when its local effects spread to the entire body via the circulation of immune cells or soluble immune mediators like cytokines, thereby causing cell and organ damage in un-infected tissues [4, 5]. However, attempts to treat sepsis by modulating circulating cytokines and their production have failed in clinical trials [6–9], and multiorgan failure of the cardiovascular, renal, pulmonary, gastrointestinal, hepatic, and hematologic systems remains a leading cause of mortality [10, 11]. International guidelines for the management of sepsis and septic shock suggest the application of antibiotics, supportive care and in the most severe cases, mechanical ventilation as well as intravenous application of vasoconstrictors and steroids [9, 12]. However, the accepted treatment strategies for ventilation, circulation and renal function do not directly address a potential dysfunction of vital central control circuits in the brain beyond symptoms of sepsis-related encephalopathy [13, 14]. Recently, however, appreciation that dysfunction of central respiratory and autonomic control may have a role in sepsis pathology has increased [15, 16].

Currently, observations indicating dysfunction of central circuits during sepsis include sympathetic hyperactivity [15, 17], altered autonomic reflexes [18] and ventilation [15, 19]. Furthermore, increases in proinflammatory cytokines in the brainstem after sterile lung injury or lipopolysaccharide (LPS) administration affect the function of central autonomic and respiratory circuits [19, 20]. Linear variability of the respiratory periodicity and nonlinear predictability of the ventilatory waveform increases in the presence of brainstem cytokine concentrations [19, 20]. These changes in the ventilatory pattern may reflect synaptic plasticity evoked by inflammatory mediators acting directly at central synapses of the vagal afferent feedback loops, as shown previously in an animal model of acute lung injury [20–22].

Our working hypothesis is that peripheral inflammation affects central autonomic circuits and consequently contributes to multiorgan failure in sepsis. We tested our hypothesis by assessing the effect of peripheral inflammation on brainstem cytokine expression and on sympatho-respiratory motor activity generated by brainstem autonomic and respiratory networks. We used an established rodent model of infectious peritonitis created by implanting a fibrin clot containing *Escherichia coli* (*E. coli*) in the abdomen [23, 24]. Compared to other animal models of sepsis, this model better mimics the spread of pathogens from an infection focus, and has been shown to reproduce the hemodynamic, metabolic alterations and cytokine response kinetics observed in patients with microbial peritonitis [24]. We found that brainstem concentrations of proinflammatory cytokines increased by 24 h after peritoneal *E. coli* implantation suggesting that infectious peritonitis evoked neuro-inflammation in brainstem autonomic and respiratory circuits. Next, we measured the functional implications of this neuro-inflammation in experiments utilizing an in situ arterially perfused brainstem preparation that generates in vivo-like patterns of sympatho-respiratory motor nerve activities in the absence of peripheral organs and blood [25, 26]. Thus, this preparation enabled us to directly assess the influence of peripheral inflammation on the brainstem circuits that control sympatho-respiratory motor patterns. In this preparation, we observed that infectious peritonitis caused an increase in perfusion pressure (an indirect measure of vascular resistance), thoracic sympathetic nerve activity, the frequency of the centrally generated respiratory rhythm and blunted the response to hypoxemic ischemia. These observations suggest that infectious peritonitis evoked synaptic plasticity in brainstem sympatho-respiratory circuits that altered sympatho-respiratory function.

## Material and methods

### Animals and ethical approval

Pathogen-free, male Sprague Dawley juvenile (P24–P27, *n* = 28) and adult rats (P90–100, *n* = 13) were housed with free access to water and food. Housing conditions were constant with a temperature of 22 °C, humidity of 60%, and a 12 h light/dark cycle. We acclimatized the rats to housing conditions for at least 5d before the experiment. The Institutional Animal Care and Use Committee at Case Western Reserve University approved the protocols for these experiments.

### Bacterial strain and fibrin clot design

The *E. coli* FDA strain was Seattle 1946 [DSM 1103, NCIB 12210] Serotype O6, Biotype 1, obtained from frozen glycerol stock (− 80 °C) that we cultured in agar medium plates and incubated overnight at 37 °C. The next day, we added one isolated colony from the plate to 10 mL tryptic soy broth and incubated overnight at room temperature. The following day, which was the day of the implantation surgery, we estimated the number of bacteria in the culture by measuring the optical density at A_600_ using a spectrophotometer. An aliquot of this *E. coli* culture was pelleted by centrifugation, washed, and suspended in saline PBS. For preparation of the fibrin clot, we mixed the suspended *E. coli* in PBS with fibrinogen (2%), and thrombin (0.015U). Sterile fibrin clots without bacteria were used as sham controls.

In adult rats (body weight, 300 g), we used *E. coli* concentrations of 75 × 10^6^ and 100 × 10^6^ colony forming units (CFU)/mL. In juvenile rats (body weight, 45–90 g), we used concentrations of 2.5 × 10^6^ and 5 × 10^6^ CFU/mL. The *E. coli* concentrations for the experiments in adult or juvenile rats were chosen because they evoked infection-dependent mortality (Additional file 1: Fig. S2). Further, all employed *E. coli* concentrations evoked an increase in respiratory frequency (see Additional file 1: Fig. SC). While infections in the clinical setting are associated with a wide variety of clinical signs and a wide range of intensities [4], in our model of microbial peritonitis which had a single pathogen that caused infection, we observed an all-or-nothing response wherein the infected rats exposed to either a low- or high-dose of *E. coli* did not significantly differ (2-way ANOVA) in either brainstem cytokine concentration (Luminex; 75–100 × 10^6^ CFU/mL, Additional file 1: Table S1) or sympatho-respiratory parameters (PBP, 2.5 to 5 × 10^6^ CFU/mL, Additional file 1: Tables S2, S3). Because of these all-or-nothing responses, we pooled the data from low- and high-doses for statistical analysis.

### Pellet implantation

The procedure for implanting a sterile or *E. coli* pellet was consistent in that we implanted fibrinogen clots in the abdomen. For both experimental adult and juvenile rats, we placed the rat supine on an aseptic surgical table. We induced and maintained anesthesia with 2% isoflurane in O_2_ at 2L/min via a customized cone mask for the duration of surgery. We monitored the rat’s body temperature and maintained it at 37 °C via a heating pad throughout the surgery. We verified the adequacy of anesthesia by the absence of a response to a toe pinch. Once the rat was unresponsive to a toe pinch, we administered a local anesthetic intramuscular injection of 2% lidocaine HCl (20 mg/mL, 0.05 mL) at the site of the abdominal incision. Then, we made a 2–3 cm midline incision in the abdomen and implanted a fibrin clot in the paracolic space. We closed the incision using a suture of 5/0 polyglactin 910 (Vicryl, Ethicon, Norderstedt, Germany) and injected meloxicam (2 mg/kg) subcutaneously to help the rats recover from surgery. Because of the experimental design of the study, we did not administer prophylactic antibiotics. We housed the rats individually and monitored their health regularly for the next 24 h. Just before the terminal experiments, we recorded the ventilatory pattern again using plethysmography (see below). In parallel, we monitored the rats’ sickness behavior and observed the following additional signs of severe infection in the infected rats: lethargy, reduced food intake, loss of body weight, poor grooming, hunched posture and porphyrin accumulation around the eyes and nose. Further, we observed a 32% mortality rate at 24 h in rats receiving a dose of 2.5 M or 5 M *E. coli* at 24 h (Additional file 1: Fig. S2) which was consistent with another study which showed a similar 24 h mortality rate and ca. 45% mortality at 48 h post-implantation [23].

### Plethysmography

We recorded the ventilatory waveform of the rats by placing them for 1 h in a flow-through, whole-body plethysmograph (Buxco Electronics, Inc., Wilmington, NC). The hour allowed the rats to acclimatize to the chamber and to rest. We recorded ventilation before and 23 h after implanting the *E. coli* (or sterile) pellet. We digitized the respiratory signals at 200 Hz sampling frequency and stored the data on a computer using commercial acquisition software (Spike2 software, Cambridge Electronic Design, Cambridge, England). Data were analyzed ‘off-line’.

### Tissue preparation for Luminex

We anesthetized the rats deeply in a chamber containing isoflurane immediately after the second plethysmography and 24 h after implanting sterile or *E. coli*-containing pellets. We lavaged the lungs and performed a craniotomy to harvest the brainstem tissue. Subsequently, we homogenized the brainstem tissue by sonication and suspended it in 200 μL of lysis buffer solution. Then, we centrifuged the solution and analyzed the supernatant for multiple antigens using a quantitative enzyme immunoassay technique (Luminex®). We analyzed the bronchoalveolar lavage fluid (BALF) and the remaining tissue homogenates using the Bradford total protein assay.

For the brainstem, we measured the concentrations of tumor necrosis factor-α (TNF-α). Interleukin-1β (IL-1β), interleukin-6 (IL-6), interleukin-17 (IL-17) and keratinocyte chemo-attractant (KC). We selected these cytokines and chemokines because they are proinflammatory and prevalent in the innate immune response, and their expression increases in the brainstem following peripheral inflammation [19–21].

### Experiments using a perfused brainstem preparation (PBP)

As with the adult rats, after the second plethysmography and 24 h after inoculation, we killed the juvenile rats to obtain in situ-perfused brainstem preparations [25, 26].

Briefly, we anesthetized rats (*n* = 28) deeply (absence withdrawal reflex) with isoflurane (5%), transected the rats below the diaphragm, immediately performed a pre-collicular decerebration and cerebellectomy, and removed the skin and lungs. We isolated (1) the left phrenic nerve and transected it at the diaphragm, (2) the left cervical vagal nerve and transected it at the supraclavicular level, and (3) the descending aorta from the ventral surface of the spinal column. Then, we transferred the preparation to the recording chamber. We cannulated the descending aorta with a double lumen cannula (1.25 mm; Braintree Scientific, Inc.). We connected a catheter to one lumen to the rat to perfuse the rat retrogradely and a pressure transducer to the other lumen to record the perfusion pressure (TA-100 transducer-amplifier, CWE Inc.). The perfusate contained (in mM): NaCl 125, KCl 3, KH_2_PO_4_ 1.25, CaCl_2_ 2.5, MgSO_4_ 1.25, NaHCO_3_ 25, D-glucose 10, and 1.25% Ficoll (an oncotic agent; Sigma‒Aldrich, Steinheim, Germany). It was gassed continuously with carbogen (95% O_2_-5% CO_2_, Air Liquid Company, Oakwood, OH), warmed to 31 °C, filtered (nylon mesh pore size: 25 µm, Millipore, Billerica, MA) and flowed through bubble traps before entering the preparation (Watson-Marlow, Wilmington, MA). Upon exiting preparation, the perfusate was collected, reoxygenated and recirculated. We maintained the flow between 22 and 40 ml/min by adjusting the number of revolutions per minute (RPM) of the peristaltic pump. Rhythmic contractions of respiratory muscles returned within 5 min after the onset of reperfusion. Adding vecuronium bromide (Hospira Inc., Lake Forest, IL) to the perfusate (0.5 mg/200 ml perfusate) blocked the contractions.

We recorded left phrenic (PNA), vagal (VNA) and thoracic sympathetic (tSNA) nerve efferent activities using suction-pipette electrodes. We amplified (Grass P511), filtered (0.1–3 kHz), digitized (10 kHz via a CED Power 1401 A/D conversion board), and stored (Dell PC with CED Spike2 software) the signals. We rectified, integrated and smoothed (50 ms time constant) the recorded signals forming ∫PNA, ∫VNA and ∫tSNA for analysis.

### Experimental protocols

We standardized the re-perfusion and experimental protocols to minimize experimental variability across PBPs. During the re-perfusion and re-oxygenation phases, we incrementally increased the revolutions of the peristaltic pump. Specifically, after cannulation of the descending aorta, we set the peristaltic pump to 30 revolution per minutes (RPM) for 5 min and then increased the rate in 10 RPM increments every 5 min until the PBPs generated the characteristic 3-phase (inspiration, post-inspiration, expiration) eupnea-like discharge pattern in PNA and VNA [26, 27]. On average, the pump rate was 70 RPM, which is a standard flow rate for PBPs from juvenile rats and produces an average of 32 mL/min.

In addition to comparing sympatho-respiratory activities in PBPs 24 h after implanting either sterile or bacterial-containing pellets, we recorded, analyzed and compared differences in the sympatho-respiratory response patterns to brief hypoxemic ischemia challenges. We created these challenges by stopping the perfusion pump for 20 s (see Fig. 3). We repeated the hypoxemic ischemia challenge 3 × and separated the challenges by 5 min. We analyzed each response and reported the average of the 3 responses.

### Analysis and statistics

From plethysmographic recordings, we analyzed respiratory cycle duration (TTOT), coefficient of variation of TTOT (CV TTOT) and respiratory frequency (fR) using 3 different 1-min epochs. The analytic methods to quantify the linear and nonlinear variability of the ventilatory pattern have been described previously [19, 20, 28].

Recording of respiratory motor activity of the PBPs allowed for the analysis of the duration of inspiration (TI, duration of PNA), post-inspiration (TPI, duration of expiratory VNA) and expiration (TE, absence of VNA at the of the expiratory interval), TTOT (onset to onset of two consecutive PNA bursts), CV TTOT and fR. We used the inspiratory onset to trigger the average 15 cycles of integrated activity. We compared cycle-triggered averages (CTAs) of ∫PNA, ∫VNA and ∫tSNA derived from rats with sterile pellets to those derived from rats with bacterially ladened pellets.

To characterize the hypoxemic ischemia response, we counted the number of respiratory cycles. For the acute response, we counted the cycles that had a TTOT shorter than the baseline TTOT (defined as the average TTOT before stopping the pump). For the post-hypoxic frequency decline, we counted the number of cycles with TTOTs longer than the baseline TTOT. For tSNA changes, we compared the magnitudes of the average ∫tSNA from the time of stopping the pump until recovery of the sham and infected groups.

Unless otherwise specified, data are presented as the mean ± standard deviation. Statistical analyses were performed using GraphPad (Prism 10). Depending on the number of independent variables and whether a variable was normally or non-normally distributed, we used an un-paired Student’s *t*-test, Mann‒Whitney *U*-test or one- or two-way ANOVA followed by a Tukey’s or Bonferroni’s multiple comparison test. Differences with a *p*-value ≤ 0.05 were considered significant.

## Results

### In vivo plethysmography pre- and post-*E. coli* infection

**Fig. 1:**
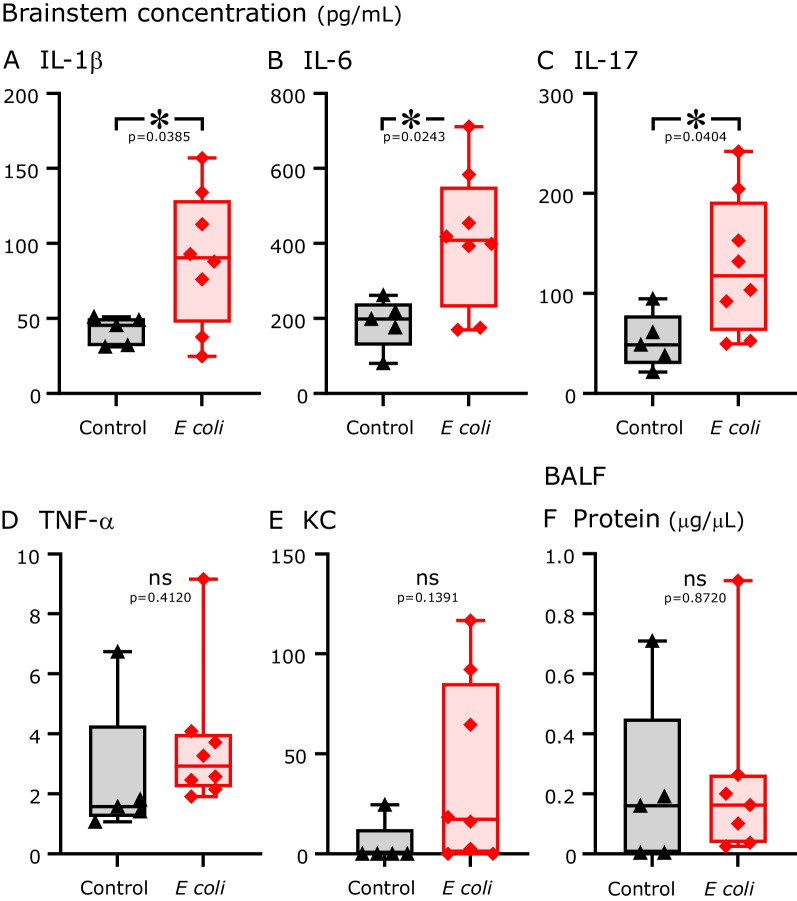
Cytokine and chemokine concentrations in the brainstem and total protein in BALF 24 h after implantation surgeries with sterile (control, black triangles) or *E. coli* pellets (red diamonds). Proinflammatory cytokines measured in the brainstem: **A** interleukin-1β (IL-1β; *p* < 0.05); **B** interleukin-6 (IL-6; *p* < 0.05); **C** interleukin-17 (IL-17; *p* < 0.05); **D** tumor necrosis factor-α (TNF-α; *n*.s.); and **E** keratinocyte chemoattractant (KC; *n*.s.). **F** Total protein concentration in BALF (*n*.s.)

At the start of each experiment, the effects of *E. coli* infection or of sterile clot implantation on respiration were monitored via in vivo plethysmography an hour before and 24 h after pellet implantation (see Additional file 1: Fig. S1). Compared to sham controls (*n* = 14), *E. coli*-infected rats (*n* = 24) displayed a significant increase in fR from 106.4 ± 24.5 to 142.8 ± 33.2 breaths/min after pellet implantation (*p* = 0.0008, Additional file 1: Fig. S1A, B, C) and no differences in the CV TTOT; Fig. 1D. However, compared to previous reports for sterile lung injury [20] or lipopolysaccharide (LPS) injection [19], we did not observe changes in linear or nonlinear ventilatory pattern variability (Additional file 1: Fig. S1E–H).

### Luminex analysis of cytokine and chemokine concentrations in the brainstem after sepsis

Luminex analysis (*n* = 8, *E. coli-*infected, *n* = 5 sham controls) revealed differences in the brainstem concentrations of proinflammatory cytokines and chemokines. *E. coli*-infected rats exhibited increases in brainstem concentrations of IL-1β, IL-6 and IL-17 compared to sham control rats (*E. coli*, 90.4 ± 44 vs. sham control, 41.7 ± 9.5 pg/mL, *p* < 0.04; *E. coli*, 413 ± 183.7 vs. sham control, 186.8 ± 67.4 pg/mL, *p* < 0.02; *E. coli*, 128.6 ± 68.8 vs. sham control, 52.7 ± 27.6 pg/mL, *p* < 0.04; respectively, Fig. 1A–C). However, the concentrations of TNFα and KC were comparable (*E. coli*, 3.7 ± 2.3 vs. sham control, 2.5 ± 2.4 pg/mL, *p* = 0.41; *E. coli*, 38.8 ± 46 vs. sham control, 4.9 ± 10.9 pg/mL, *p* = 0.14, Fig. 1D–E). The total protein concentration of BALF did not differ (*E. coli* [*n* = 7], 0.24 ± 0.3 vs. sham control [*n* = 5], 0.21 ± 0.3 µg/µL, *p* = 0.87, Fig. 1F).

### Functional changes in septic in situ-perfused brainstem preparations

**Fig. 2:**
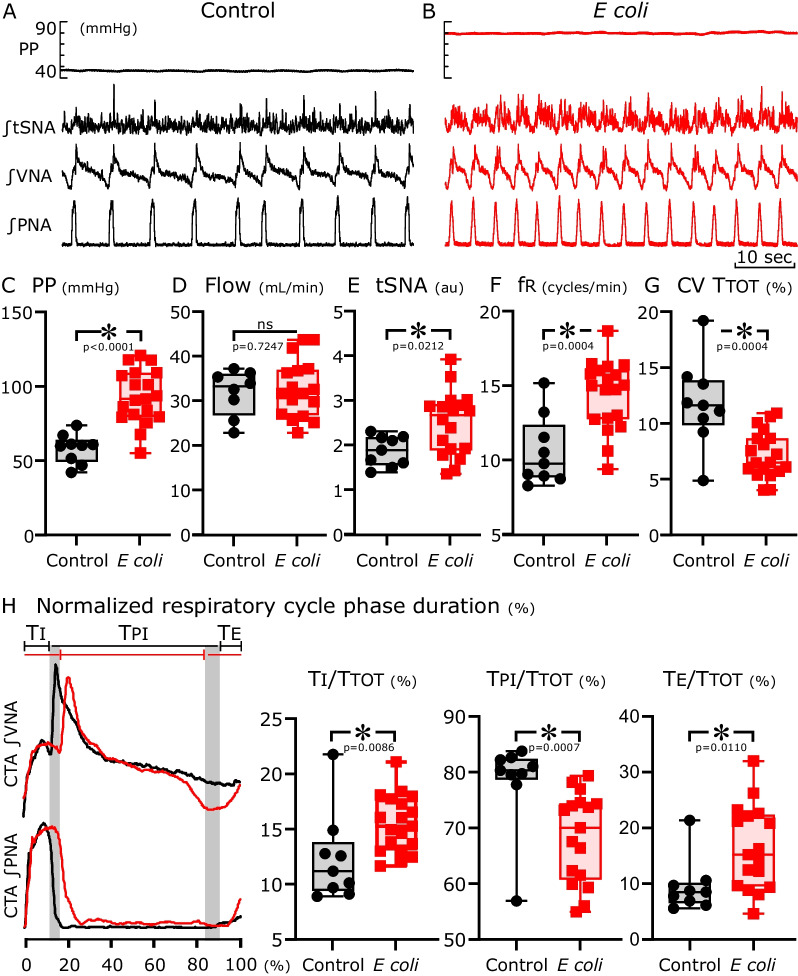
Respiratory motor pattern from *n* = 24 in situ preparations 24 h after pellet implantation. **A**, **B** Representative recordings (from top to bottom) of the perfusion pressure (PP), integrated thoracic sympathetic chain (tSNA), vagal (VNA) and phrenic (PNA) nerve activity from preparations obtained from animals of the sham control group (black) or the *E. coli*-infected group (red). **C–G** Group data for the mean perfusion pressure (**C**); flow (**D**); integrated t-SNA (**E**); respiratory burst frequency (fR, **F**) and coefficient of variation for total respiratory cycle length (CV TTOT, G) from preparations obtained from sham controls and from *E. coli*-infected animals. **H** Comparison of cycle-triggered averages (CTA) of integrated vagal and phrenic activities of sham control (black dashed line) and *E. coli* (red line)-infected animals. The left panel highlights the changes (gray shaded areas) in the respiratory phase durations (TI = time of inspiration; TPI = time of post-inspiration (phase 1 expiration) and TE = time of expiration (phase 2 expiration)) in relation to the normalized respiratory cycle length

Analysis of sympatho-respiratory activity generated by the brainstem in PBPs revealed differences between preparations from uninfected rats (Fig. 2A) and those from rats with sepsis (Fig. 2B). Infected PBPs had increased perfusion pressure (*E. coli*, 93.1 ± 18.1 vs. sham control, 58.2 ± 9.8 mmHg, *p* < 0.0001, Fig. 2C), even though flow did not differ in either group (Fig. 2D). Normalized ∫tSNA across the respiratory cycle increased (*E. coli*, 2.49 ± 0.71 vs. sham control, 1.87 ± 0.34 a.u., *p* < 0.03, Fig. 2E). fR increased and the CV TTOT decreased in *E. coli*-treated PBPs compared to the sham control (*E. coli*, 14.37 ± 2.3 vs. sham control, 10.57 ± 2.3 respiratory cycles/min, *p* < 0.0001, Fig. 2F and * E. coli*, 7.19 ± 2.1 vs. sham control, 11.78 ± 3.9%, *p* < 0.0001, Fig. 2G). The increased fR was due to a decrease in the duration of post-inspiration (TPI) because the durations of inspiration (TI) and expiration (TE) relative to TTOT increased (Fig. 2H p < 0.009; *p* < 0.01, *p* < 0.0007, respectively).

### Response to hypoxemic ischemia

**Fig. 3:**
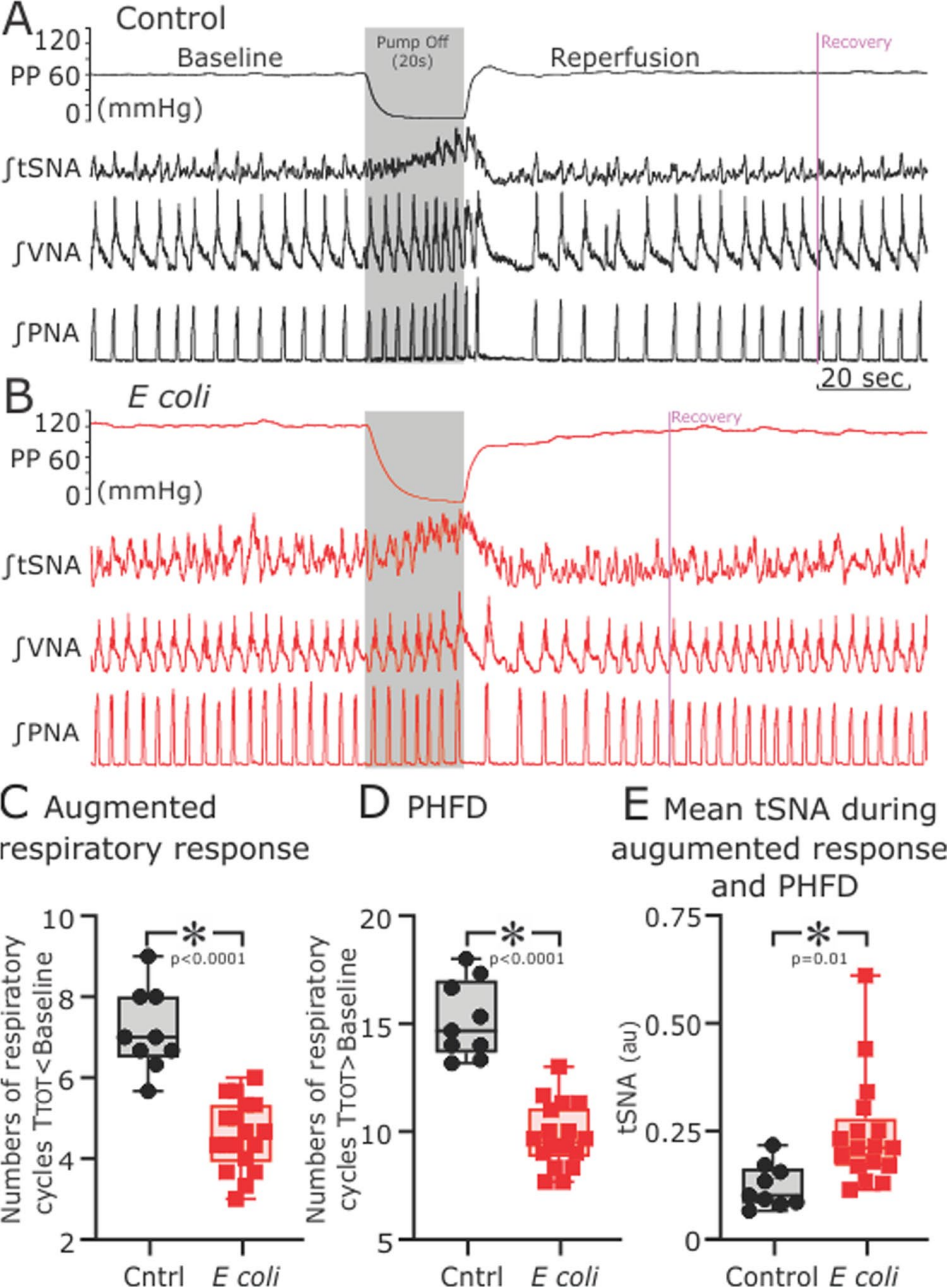
Brief hypoxemic ischemia challenges reveal changes in sympatho-respiratory response in relation to peripheral infection. Representative recordings of perfusion pressure (PP), integrated thoracic sympathetic chain (tSNA), vagal (VNA) and phrenic (PNA) nerve activity from sham control (**A** black traces) and *E. coli*-infected preparations (**B** red traces) before and after brief hypoxemic ischemia, mimicked by stopping the perfusion pump for 20 s. Compared to sham controls, we observed a decrease in the strength of the initial hypoxemic ischemia respiratory augmentation (**C** lower number of respiratory cycles with TTOT shorter than TTOT at baseline before stopping the pump in the *E. coli* group); a significantly less pronounced post-hypoxic frequency decline (**D** lower number of respiratory cycles with TTOT longer than TTOT at baseline before stopping the pump in the *E. coli* group) accompanied by significantly enhanced integrated tSNA (**E**)

We tested the response to brief hypoxemic ischemia challenges by stopping the perfusion pump for 20 s (Fig. 3). This evoked a biphasic respiratory response of an initial increase in the PNA burst frequency followed by a post-hypoxemic ischemia PNA burst frequency decline in both sham controls (Fig. 3A) and *E. coli*-infected preparations (Fig. 3B). The number of PNA bursts that had a shorter (augmentation phase) or longer TTOT (post-hypoxemic ischemia frequency decline) compared to TTOT at baseline decreased in *E. coli-*infected PBPs (*E. coli*, 4.57 ± 0.85 vs. sham control, 7.15 ± 1.02 PNA bursts, *p* < 0.0001, Fig. 3C and * E. coli*, 7.57 ± 1.21 vs. sham control, 14.78 ± 1.68 bursts, *p* < 0.0001, Fig. 3D).

Although the respiratory response to hypoxemic ischemia was attenuated in *E. coli*-infected animals, the sympathetic drive (tSNA) from the onset of ischemia (pump off) until recovery of baseline fR was significantly enhanced (*E. coli*, 0.25 ± 0.12 vs. sham control, 0.12 ± 0.05 au, *p* < 0.02, Fig. 3E).

## Discussion

In this study, we showed that bacterial peritonitis triggered an increase in proinflammatory cytokine levels in the brainstem that were correlated with the altered function of the central sympatho-respiratory control circuits. These changes consisted of both an increased respiratory frequency in intact rats and an increase in the brainstem-generated sympatho-respiratory motor drive in in situ PBP at baseline. Moreover, PBP revealed pathophysiological changes in response to hypoxemic ischemia, which can be attributed to inflamed peripheral receptor systems and neuronal plasticity in the brainstem circuits that regulate autonomic and respiratory patterns.

The central nervous system effects of sepsis include delirium and sickness behavior [14, 29, 30], whereas the potential effects of peripheral inflammation on sympatho-respiratory circuits in the brainstem have been previously considered to predominantly occur via altered reflex feedback arising from arterial baroreceptors, pulmonary stretch receptors and chemoreceptors of the carotid body [16, 18]. Previous work from our lab explored the role of brainstem inflammation in disease models such as LPS [19] and sterile lung injury [20–22]. Acute lung injury causes an increase in cytokine expression in the area postrema and nucleus of the solitary tract (sensory relay nucleus for pulmonary afferents) as well as changes in brainstem respiratory network function due to inflammation-dependent changes in pulmonary receptor feedback [20–22].

In the present study, we investigated the effect of bacterial-evoked sepsis on brainstem-controlled sympatho-respiratory function in a clinically relevant animal model of peritonitis [24]. Like inflammation due to sterile lung injury [20–22] and repetitive LPS injections [19], peripherally induced inflammation in our rat model of sepsis triggered an increase in the concentration of the proinflammatory cytokines IL-1β, IL-6 and IL-17 in the brainstem. Further, similar to peripheral inflammation evoked by sterile lung injury [20], sepsis evoked changes in brainstem-generated breathing motor nerve activities in perfused brainstem preparations, including an increase in respiratory frequency (fR) and reduced variability in respiratory cycle length (CV TTOT). Further, peritonitis also evoked a subtle shift in the relative phase duration in the three-phase respiratory motor pattern at baseline.

We showed, for the first time, that peritonitis causes changes in the central sympathetic motor drive during baseline and during hypoxemic ischemia. At baseline, we observed sustained tSNA in the presence of high perfusion pressure. This represents an increase in vascular resistance because flow did not differ between preparations. Thus, it can be attributed to an increase in sympathetic vasomotor tone because potential effects of circulating cytokines and hormones can be excluded (removal of kidneys, adrenals and other glands, exsanguination). The observed effect at baseline may underestimate the increase in sympathetic drive since elevated perfusion pressure in the PBP activates the baroreceptor reflex which substantially decreases sympathetic activity [26, 31]. Thus, the results of the present study suggest that *E. coli* infection causes neurogenic hypertension [32].

Proinflammatory cytokines are potent neuromodulators of brainstem circuits involved in the generation of sympathetic motor drive and thereby may be key players in the neurogenic hypertension we observed [33, 34]. For instance, IL-1β and IL-6 can increase sympathetic nerve activity when applied centrally [35, 36]. Centrally applied IL-6 decreases baroreceptor reflex sensitivity [34] and thus may have contributed to the mechanism of increasing sympathetic activity to maintain perfusion pressure in the presence of systemic *E. coli* infection.

In vivo, global vasodilation is a hallmark of severe sepsis [37]. Our results in the in situ-perfused brainstem preparation suggest that sepsis-related hypotension may relate more to humoral factors in the blood and local control of the vascular bed than to brainstem sympathetic/parasympathetic networks. Clinically, hypotension is treated with the application of vasoconstrictors, which may further exaggerate a sympathetic–parasympathetic imbalance in sepsis and may further contribute to multiorgan failure [38], particularly acute kidney failure [39]. The augmentation of the sympathetic response and the attenuation of the respiratory response during hypoxemic ischemia further illustrates that peripheral inflammation can cause brainstem dysfunction in circuits that may directly contribute to multiorgan failure in sepsis.

Mechanistically, the changes observed in the arterially perfused brainstem preparation likely reflect synaptic plasticity in brainstem autonomic and respiratory circuits evoked by peripheral inflammation in our model of microbial sepsis. The arterially perfused brainstem preparation is an ideal model to investigate brainstem-mediated autonomic and respiratory control because it maintains the anatomical and functional integrity of brainstem circuits [25, 26]. Moreover, circuit activity is not modulated by sensory feedback from the periphery or humoral factors in the blood, has a constant metabolic (CO_2_) drive, and lacks the confound of anesthesia since it is decerebrate [25, 26]. A key component of our study design is that blood-borne components of the immune system were removed and replaced with aCSF that lacks these factors. Thus, the sympathetic and respiratory nerve activities monitored in the present study reflect only the brainstem-circuit mechanisms that maintain their rhythm and pattern. As such, we interpret differences between PBPs from sham controls versus PBPs from *E. coli*-infected rats to indicate synaptic plasticity in brainstem autonomic and respiratory circuits that was evoked by peritoneal infection. It is well known that such long-term synaptic plasticity can occur on time scales as short as seconds or over longer time scales of minutes to hours [40, 41]. In the brainstem respiratory network, it has been established that Hebbian synaptic plasticity can occur within minutes in response to vagal nerve stimulation [42, 43]. Thus, it is plausible that within 24 h, microbial sepsis evoked lasting synaptic changes in brainstem autonomic and respiratory circuits that manifest as changes in sympathetic and respiratory nerve activities. An interesting topic of future investigation will be whether cytokines in the brainstem play a role in mediating synaptic plasticity in brainstem autonomic and respiratory circuits in sepsis.

## Conclusion

The present study demonstrates that *E. coli* peritonitis triggers significant changes in brainstem sympatho-respiratory control circuits and opens a new chapter for the understanding and treatment of life-threatening multiorgan failure in sepsis.

### Supplementary Information

**Additional file 1: Fig S1.** Changes in the in vivo ventilatory pattern before and after pellet implantation. A-B Representative plethysmography recordings of ventilatory activity in sham control (black) and *E. coli*-infected animals (red) at baseline (1 h before pellet implantation) and 24 h after pellet implantation illustrate that respiratory frequency only increased in animals with *E. coli* infection (see group data in C), while the coefficient of variation of the respiratory cycle length (CV TTOT) remained unchanged in both experimental groups (see group data in D). We also did not detect any significant difference in either the linear (autocorrelation, mutual information and sample AC, E) or nonlinear complexity index of the ventilatory pattern (NLCI, F). **Fig S2.** Survival post-implantation of sterile or E. coli-inoculated fibrin clots in the abdomen. Sham control rats (*n* = 7) showed 100% survival after implantation of a sterile fibrin clot. Both rats that received a dose of 2.5 M (*n* = 12) or 5 M E. coli (*n* = 12) via the fibrin clot showed a 32% mortality rate at 24 h. Note that we added some jitter to the E. coli-infected groups for visualization. **Table S1.** One-way analysis of variance (ANOVA) comparing mean cytokine concentration values from brainstem and total protein value from bronchoalveolar lavage fluid (BALF) of 75 × 10^6^ *E. coli* or 100 × 10^6^ *E. coli* 24 h after pellet implantation. In all cases, there were no significant differences between the two doses of E. coli. Data are shown as mean ± SEM. Abbreviations: IL-1β, Interleukine-1β; IL-6, Interleukine-6; IL-17, Interleukine-17; KC, keratinocyte chemoattractant, TNFα, tumor necrosis factor α; Protein: Protein in broncho-alveolar lavage fluid. **Table S2.** One-way analysis of variance (ANOVA) comparing baseline sympatho-respiratory parameters measured from PBP recordings of rats infected with 2.5 × 10^6^ *E. coli* or 5 × 10^6^ *E. coli* 24 h after pellet implantation. In all cases, there were no significant differences between the two doses of E. coli. Data are shown as mean ± SEM. Abbreviations: PP, perfusion pressure; Flow, flow of the perfusate (aCSF); tSNA, thoracic sympathetic nerve activity; fR, respiratory frequency; CV TTOT, coefficient of variation for total cycle length; TI, inspiratory duration; TPI, post-inspiratory duration; TE, expiratory phase 2 duration, TTOT, total respiratory cycle duration. **Table S3.** One-way analysis of variance (ANOVA) comparing sympatho-respiratory parameters measured from PBP recordings of rats infected with 2.5 × 10^6^ *E. coli* or 5 × 10^6^ *E. coli* during hypoxemic ischemia challenges. In all cases, there were no significant differences between the two doses of E. coli. Data are shown as mean ± SEM. Abbreviations: ARR, augmented respiratory response; PHFD, post-hypoxic frequency decline; tSNA/time, mean integrated thoracic sympathetic nerve activity per second.

## Data Availability

The datasets used during the current study are available from the corresponding author upon reasonable request.
