# Supplementary material for: Peritoneal sepsis caused by Escherichia coli triggers brainstem inflammation and alters the function of sympatho-respiratory control circuits
Source: J Neuroinflammation. 2024 Feb 8;21:45. doi: 10.1186/s12974-024-03025-7 (PMC10854125; doi:10.1186/s12974-024-03025-7)
Supplement: Supplementary file 1 — Additional file 1: Fig S1. Changes in the in vivo ventilatory pattern before and after pellet implantation. A-B Representative plethysmography recordings of ventilatory activity in sham control (black) and E. coli-infected animals (red) at baseline (1 h before pellet implantation) and 24 h after pellet implantation illustrate that respiratory frequency only increased in animals with E. coli infection (see group data in C), while the coefficient of variation of the respiratory cycle length (CV TTOT) remained unchanged in both experimental groups (see group data in D). We also did not detect any significant difference in either the linear (autocorrelation, mutual information and sample AC, E) or nonlinear complexity index of the ventilatory pattern (NLCI, F). Fig S2. Survival post-implantation of sterile or E. coli-inoculated fibrin clots in the abdomen. Sham control rats (n = 7) showed 100% survival after implantation of a sterile fibrin clot. Both rats that received a dose of 2.5 M (n = 12) or 5 M E. coli (n = 12) via the fibrin clot showed a 32% mortality rate at 24 h. Note that we added some jitter to the E. coli-infected groups for visualization. Table S1. One-way analysis of variance (ANOVA) comparing mean cytokine concentration values from brainstem and total protein value from bronchoalveolar lavage fluid (BALF) of 75 × 106 E. coli or 100 × 106 E. coli 24 h after pellet implantation. In all cases, there were no significant differences between the two doses of E. coli. Data are shown as mean ± SEM. Abbreviations: IL-1β, Interleukine-1β; IL-6, Interleukine-6; IL-17, Interleukine-17; KC, keratinocyte chemoattractant, TNFα, tumor necrosis factor α; Protein: Protein in broncho-alveolar lavage fluid. Table S2. One-way analysis of variance (ANOVA) comparing baseline sympatho-respiratory parameters measured from PBP recordings of rats infected with 2.5 × 10^6^ E. coli or 5 × 10^6^ E. coli 24 h after pellet implantation. In all cases, there were no significant differences [file 12974_2024_3025_MOESM1_ESM.docx]

**Additional File for**


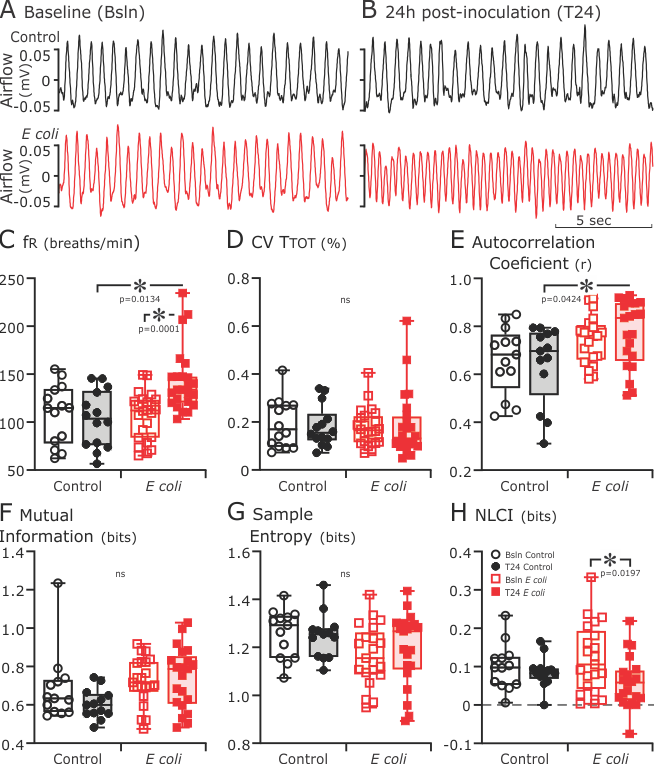


**Fig S1. Changes in the *in vivo* ventilatory pattern before and after pellet implantation.**

**A-B** Representative plethysmography recordings of ventilatory activity in sham control (black) and *E. coli*-infected animals (red) at baseline (1 hour before pellet implantation) and 24 hours after pellet implantation illustrate that respiratory frequency only increased in animals with *E. coli* infection (see group data in **C**), while the coefficient of variation of the respiratory cycle length (CV TTOT) remained unchanged in both experimental groups (see group data in **D**). We also did not detect any significant difference in either the linear (autocorrelation, mutual information and sample AC, **E**) or nonlinear complexity index of the ventilatory pattern (NLCI, **F**).


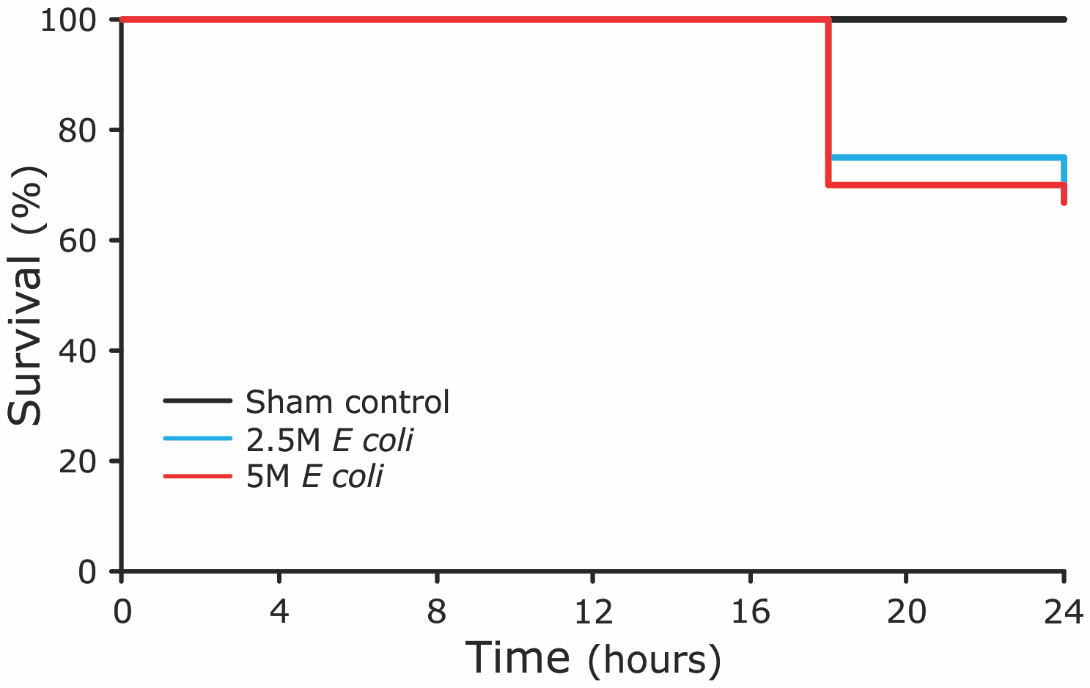


**Fig S2. Kaplan-Meier Survival curves of observed the mortality.**

The infection was severe enough to cause a 40% mortality within 24h in rats implanted with E. coli-containing clots. There was no mortality in those rats implanted with a sterile-fibrin clot. Given that 40% mortality is comparable to that observed clinically and that the rats that survived to 24h appeared to be very ill, we conclude that the changes in the central control of autonomic and respiratory circuit function became maladaptive by 24h.

| **Variable** | **75M *E. coli*** | **100M *E. coli*** | **P value** |
| --- | --- | --- | --- |
| IL-1β | 88.15 ± 19.03 | 92.63 ± 19.03 | 0.985 |
| IL-6 | 400.17 ± 79.53 | 425.88 ± 79.53 | 0.972 |
| IL-17 | 128.39 ± 30.07 | 128.83 ± 30.07 | 1.000 |
| KC | 45.97 ± 19.31 | 31.68 ± 19.31 | 0.862 |
| TNFα | 2.99 ± 1.20 | 4.34 ± 1.20 | 0.713 |
| Protein | 0.091 ± 0.138 | 0.446 ± 0.159 | 0.263 |

**Table S1** One-way analysis of variance (ANOVA) comparing mean cytokine concentration values from brainstem and total protein value from bronchoalveolar lavage fluid (BALF) of 75x10^6^ *E. coli* or 100x10^6^ *E. coli* 24 hours after pellet implantation. In all cases, there were no significant differences between the two doses of E. coli. Data are shown as mean ± SEM. Abbreviations: IL-1β, Interleukine-1β; IL-6, Interleukine-6; IL-17, Interleukine-17; KC, keratinocyte chemoattractant, TNFα, tumor necrosis factor α; Protein: Protein in broncho-alveolar lavage fluid.

| **Variable** | **2.5M *E coli*** | **5M *E coli*** | **P value** |
| --- | --- | --- | --- |
| PP | 91.631 ± 5.423 | 94.385 ± 5.144 | 0.928 |
| Flow | 31.058 ± 2.311 | 33.831 ± 1.933 | 0.633 |
| tSNA | 2.506 ± 0.211 | 2.468 ± 0.211 | 0.991 |
| fR | 14.793 ± 0.777 | 13.984 ± 0.737 | 0.733 |
| CV TTOT | 6.886 ± 0.937 | 7.461 ± 0.889 | 0.897 |
| TI/TTOT | 16.264 ± 1.029 | 14.809 ± 0.976 | 0.567 |
| TPI/TTOT | 67.677 ± 3.108 | 68.844 ± 2.600 | 0.955 |
| TE/TTOT | 16.411 ± 2.589 | 16.346 ± 2.166 | 1.000 |

**Table S2** One-way analysis of variance (ANOVA) comparing baseline sympatho-respiratory parameters measured from PBP recordings of rats infected with 2.5x10^6^ *E. coli* or 5x10^6^ *E. coli* 24 hours after pellet implantation. In all cases, there were no significant differences between the two doses of E. coli. Data are shown as mean ± SEM. Abbreviations: PP, perfusion pressure; Flow, flow of the perfusate (aCSF); tSNA, thoracic sympathetic nerve activity; fR, respiratory frequency; CV TTOT, coefficient of variation for total cycle length; TI, inspiratory duration; TPI, post-inspiratory duration; TE, expiratory phase 2 duration, TTOT, total respiratory cycle duration.

| **Variable** | **2.5M *E coli*** | **5M *E coli*** | **P value** |
| --- | --- | --- | --- |
| ARR | 4.852 ± 0.297 | 4.296 ± 0.297 | 0.396 |
| PHFD | 7.630 ± 0.470 | 7.519 ± 0.470 | 0.985 |
| tSNA/s | 0.289 ± 0.036 | 0.205 ± 0.034 | 0.219 |

**Table S3** One-way analysis of variance (ANOVA) comparing sympatho-respiratory parameters measured from PBP recordings of rats infected with 2.5x10^6^ *E. coli* or 5x10^6^ *E. coli* during hypoxemic ischemia challenges. In all cases, there were no significant differences between the two doses of E. coli. Data are shown as mean ± SEM. Abbreviations: ARR, augmented respiratory response; PHFD, post-hypoxic frequency decline; tSNA/time, mean integrated thoracic sympathetic nerve activity per second.
